# Qualities of Culturally and Religiously Sensitive Practice: A Cross-Sectional Study

**DOI:** 10.1177/08258597211050742

**Published:** 2021-11-29

**Authors:** Panagiotis Pentaris, Panayiota Christodoulou

**Affiliations:** 1 School of Human Sciences, Institute for Lifcourse Development, University of Greenwich, London, UK; 2 Department of Psychology and Social Sciences, Frederick University, Limassol, Cyprus

**Keywords:** culture, religion, palliative care, hospice, Cyprus

## Abstract

**Background:** Culture and religion influence lived experience and particularly dying and grieving. Research has largely focused on exploring culturally and religiously sensitive practices, but not necessarily in palliative and hospice care or across nations. Acquired knowledge from the more advanced end-of-life care systems (eg the UK) tends to be generalized to other contexts where its cultural appropriation is not tested. **Aim:** This study explored the different qualities, among hospice and palliative professionals in Cyprus, describing cultural competence, cultural humility, and religious literacy. **Design:** A cross-sectional study of 41 palliative and hospice professionals in Cyprus, with the use of a 5-point Likert style questionnaire (*a* = 0.898). **Setting:** The study took place in Cyprus and participants were recruited from across palliative and hospice care organizations, including the only hospice in Cyprus, Cyprus Association of Cancer Patients and Friends (PASYKAF), and the Cyprus Anti-Cancer Society (CACS). **Results:** This study found that there are four main qualities that lead to effective culturally and religiously sensitive practice—informed decision-making, respect, adaptability, and nonjudgmental practice. **Conclusions:** Future education and training of professionals can consider these findings to appropriate approaches in practice that fit the Cypriot end-of-life care context more effectively.

## Introduction

Culturally and religiously sensitive practice has been debated for over three decades.^1^ Professionals acknowledged the necessity for a more diverse approach as the cultural and religious aspects of clients/patients directly influence the effectiveness of their treatment,^1^ as well as inform decision-making processes.^2^ Albeit the breadth of information in current literature about sensitive practice, a more standardized descriptor of what constitutes appropriate culturally and religiously sensitive practice remains unexplored or ambiguous at best. An example that examines this includes Al-Krenawi and Graham^3^ who suggested that taking into account a client’s gender relations, place in their family and community (status), and level of acculturation when the individual is a migrant, is crucial when attempting a culturally sensitive practice. Another example is Danso,^4^ whose analysis is drawing on various descriptors and argues the need for cultural humility—a process of inter- and intrapersonal learning whereby a growing understanding of own beliefs and identities is crucial—and the need for heightened levels of understanding of one’s cultural makeup. Such studies have added critically to our appreciation of how to achieve culturally sensitive practice but have primarily focused on mental health services and health care altogether, but not palliative and hospice or religious sensitive work care necessarily.

The present study explores the qualities of culturally and religiously sensitive practices of professionals working in palliative care and hospice settings in Cyprus. Cyprus, as a country, is fairly new to the concept of diversity in general and racism and discrimination are often observed within the Cypriot society. That said, this study aimed to explore professionals’ perceptions about their patients’ diverse characteristics and whether those perceptions interfere with the effectiveness of their interventions with them. The researchers also aimed to explore whether the professionals were equipped with the necessary skills to manage patients from different cultures and/or religions.

## Methodology

This report is part of a larger, cross-sectional study that explored the wide range of knowledge, skills, and attitudes of hospice and palliative professionals in Cyprus, pertaining to cultural competence, cultural humility, and religious literacy. The present part of the study focused on the varied characteristics consistent in sensitive practice vis-à-vis culture, religion, and spiritual identities in hospice and palliative care. Further, the study received ethical approval from the Cyprus National Bioethics Committee (EEBK EP 2019.01.28).

Altogether, the study took place in Cyprus (Greek Cypriot controlled southern two-thirds of the island), a small nation with consistent maintenance of services in the care of cancer patients since the 1970s, but little advancements in end-of-life care on the whole. Currently, the nation merely presents 1 hospice for adult care, with 25 beds, while only a few professionals are occupied in hospice and palliative care, albeit end-of-life care practices in general health care.

### Sample and Sampling Method

A combination of purposive and snowballing techniques (9) was used. A call for participants was circulated via key organizations and networks in Cyprus; namely, the not-for-profit organizations: Cyprus Anti-Cancer Society (CACS) and Cyprus Association of Cancer Patients and Friends (PASYKAF), as well as the Cyprus Muscular Dystrophy Association, the Cyprus Institute of Neurology and Genetics, Cyprus Social Work Association, Cyprus Psychologists Associations, and Network of Hospice and Palliative Care Professionals in Cyprus. Religious organizations or others that does not employ hospice and palliative professionals were not approached during the recruitment phase. Individuals who showed interest were screened for eligibility criteria (ie registered health and/or social care professionals engaging with hospice and palliative care practices with their professional role) provided with participant information and the opportunity to ask further questions of the researchers. Once consent was received, participants completed a Qualtrics-based self-administered survey of the measurement tool described below.

### Measurement Tool

Drawing on the Greater Vancouver Island Multicultural Society Cultural Competence Self-Assessment Checklist (Greater Vancouver Island Multicultural Society at www.cvims.org), which consists of 33 items divided into 3 categories (ie knowledge, awareness, and skills), a new tool was devised. Specifically, adjustments to the tool included the alignment of the concepts of aboriginality, to better fit the context; and change of reference to Canadian history and replaced with Cypriot history. A 5-point Likert style questionnaire was produced by the researchers, measuring answers from *never* (1) to *always* (5).^5^ The tool was reviewed by an academic and a practitioner in the field, who confirmed the statements’ relevance and significance. The newly devised questionnaire was also piloted with 10 professionals, which equally increased its validity. Cronbach’s alpha coefficient (*a* = 0.898) of the questionnaire showed high consistency, while a Cronbach’s alpha of the third part of the questionnaire (ie skills), exploring the qualities of sensitive practice in end-of-life care, was 0.915, showing very high consistency of the tool.

### Data Analysis

The data were inserted into SPSS version 25 and univariate and bivariate analyses were carried out. Frequencies were calculated to identify areas of significance in the data; correlations (Pearson’s) assisted in recognizing the varied associations between variables and those within the segment of the questionnaire focusing on skills. Further, regression analysis was carried out to explore the variations of variables when associated with others.

## Results

A total of 53 questionnaires were returned, which is approximately 60% of all hospice and palliative professionals in Cyprus (this excludes professionals in general health care who might in their role engage with end-of-life matters). Of the 53 questionnaires, 12 were missing values and were excluded, resulting in N = 41 participants altogether. The majority were female (68.3%), between the ages of 25 and 34 years (61%), Greek Cypriot (90.2%), Orthodox (85.4%), and with a postgraduate education (58.5%). Table 1 paints a clearer picture of the participants and helps appreciate the way the findings are interpreted in the context.

**Table 1.** Participants’ Characteristics.

Variable		% (N = 41)	Mean (SD)
Gender	Male	31.7%	–
	Female	68.3%	
Age (years)	25 to 34	61%	30 (0.865)
	35 to 44	24.4%	
	45 to 54	9.8%	
	55 to 64	4.9%	
Nationality	Cypriot	90.2%	− (0.3)
	Greek	9.8%	
Religion/Nonreligion	Orthodox	85.4%	–
	Catholic	2.4%	
	Buddhist	2.4%	
	Atheist	7.3%	
	Spiritual	2.4%	
Family structure	Single	32.5%	(1.24)
	Married/in partnership	50%	
	Divorced/separated	5%	
	Cohabiting	12.5%	
Education	College (IEK)	2.4%	–
	Undergraduate	31.7%	
	Postgraduate	58.5%	
	PhD/Doctorate	4.9%	
	Postdoctorate	2.4%	
Discipline	Nurse	4.9%	–
	Physician/Doctor	4.9%	
	Social worker	56.1%	
	Psychologist	17.1%	
	Occupational therapist	7.3%	
	Priest	2.4%	
	Other	7.3%	
Sector	Public	22%	–
	Private	36.6%	
	Voluntary	34.1%	
	Other	7.3%	
Income	<€15,000	31.7%	€16,000 to €25,000 (0.961)
	€16,000 to €25,000	46.3%	
	€26,000 to €35,000	9.8%	
	>€36,000	12.2%	
Overall experience	0 to 2 years	10.8%	11 to 20 years (1.2)
	3 to 5 years	16.2%	
	6 to 10 years	24.3%	
	11 to 20 years	37.8%	
	21 to 30 years	8.1%	
	31 to 40 years	2.7%	
Experience in palliative and hospice care	0 to 2 years	38.9%	3 years (1.1)
	3 to 5 years	27.8%	
	6 to 10 years	16.7%	
	11 to 20 years	16.7%	

### Inferential Statistics

A Pearson’s analysis revealed both positive and negative relationships between a number of variables. This analysis helps appreciate the complex tendencies found in the data, which illustrate the characteristics of culturally and religiously sensitive practice, as well as the prerequisites for improving or enhancing them. Table 2 depicts the correlations between all items in the questionnaire that pertain to skills and knowledge about cultural competence and religious literacy in end-of-life care; yet, only those reporting on sensitive practice around culture and religion are examined for the purposes of this paper (in bold in Table 2). Table 3 identifies the statements matching the codes in the former table.

**Table 2.** Pearson’s Correlation Coefficient of Skills Variables Pertaining to Sensitive Practice.

		QE3	QE5	QE6	QE9	QE11	QG4	QG7	QG10	QG11	QG13	QD1	QD3	QD6	QD10	QD11	QD12
QE3	Pearson’s Correlation	1															
	Sig. (two-tailed)																
QE5	Pearson’s Correlation	0.278	1														
	Sig. (two-tailed)	0.106															
QE6	Pearson’s Correlation	.356*	.406*	1													
	Sig. (two-tailed)	0.033	0.015														
QE9	Pearson’s Correlation	0.272	.436*	0.119	1												
	Sig. (two-tailed)	0.125	0.013	0.508													
QE11	Pearson Co’srrelation	0.097	0.216	0.108	0.057	1											
	Sig. (two-tailed)	0.593	0.235	0.550	0.752												
QG4	Pearson’s Correlation	0.279	.452**	.648**	.380*	0.232	1										
	Sig. (two-tailed)	0.110	0.008	0.000	0.029	0.193											
QG7	Pearson’s Correlation	0.106	−0.146	0.277	0.062	.403*	0.126	1									
	Sig. (two-tailed)	0.571	0.440	0.132	0.745	0.027	0.501										
QG10	Pearson’s Correlation	0.017	0.033	0.315	.388*	0.141	.389*	.360*	1								
	Sig. (two-tailed)	0.929	0.861	0.085	0.034	0.457	0.030	0.047									
QG11	Pearson’s 'Correlation	0.284	.429*	.511**	0.197	0.105	.490**	0.021	.392*	1							
	Sig. (two-tailed)	0.122	0.018	0.003	0.297	0.580	0.005	0.911	0.029								
QG13	Pearson’s Correlation	.447*	0.155	0.181	0.308	.391*	0.246	.655**	.386*	−0.032	1						
	Sig. (two-tailed)	0.017	0.439	0.357	0.118	0.043	0.207	0.000	0.042	0.872							
QD1	Pearson’s Correlation	−0.058	0.355	0.224	.581**	.402*	.442*	0.214	.516**	0.064	0.348	1					
	Sig. (two-tailed)	0.770	0.069	0.252	0.001	0.038	0.018	0.275	0.005	0.746	0.070						
QD3	Pearson’s Correlation	0.039	.535**	.383*	.439*	0.115	.702**	−0.231	0.223	0.354	−0.052	.400*	1				
	Sig. (two-tailed)	0.843	0.004	0.044	0.022	0.569	0.000	0.237	0.255	0.065	0.791	0.035					
QD6	Pearson’s Correlation	**-**.375*	0.152	0.000	0.229	0.042	0.155	0.054	.500**	0.239	−0.047	.515**	0.357	1			
	Sig. (two-tailed)	0.050	0.448	1.000	0.250	0.834	0.432	0.784	0.007	0.221	0.813	0.005	0.062				
QD10	Pearson’s Correlation	−0.176	.464*	−0.067	.505**	0.288	0.129	−0.034	0.234	0.071	−0.039	.580**	0.321	.672**	1		
	Sig. (two-tailed)	0.371	0.015	0.734	0.007	0.145	0.513	0.864	0.230	0.720	0.844	0.001	0.096	0.000			
QD11	Pearson’s Correlation	−0.279	.432*	−0.034	0.252	.424*	0.038	−0.040	−0.046	−0.056	−0.139	.575**	0.173	.413*	.723**	1	
	Sig. (two-tailed)	0.151	0.024	0.863	0.204	0.027	0.847	0.839	0.815	0.777	0.480	0.001	0.378	0.029	0.000		
QD12	Pearson’s Correlation	−0.181	.507**	0.019	0.243	0.240	0.009	−0.043	0.057	0.169	−0.102	.445*	0.087	.577**	.756**	.689**	1
	Sig. (two-tailed)	0.357	0.007	0.922	0.221	0.228	0.962	0.830	0.775	0.391	0.606	0.018	0.659	0.001	0.000	0.000	

*Correlation is significant at the 0.05 level (two-tailed).

**Correlation is significant at the 0.01 level (two-tailed).

**Table 3.** Items with Descriptions.

Code/Item	Description	Mean	SD
QE3	In order to enhance own knowledge about others’ culture and religion, becoming fully aware of own identities and sharing that knowledge is essential	3.62	.57
QE5	I am fully aware of all implicit and explicit assumptions I make about people with a different cultural or religious identity than mine	3.12	.71
QE6	My own cultural and religious beliefs influence professional judgment and decisions, as well as a structured notion of “normality”	3.31	.62
QE9	I seize all opportunities to engage with people from different backgrounds, in order to increase my knowledge and build rapport	2.81	.69
QE11	I appreciate that challenges in the social life of the host nation may further impact on the lives of migrants or people with different than the normative identities	3.35	.63
QG4	I appreciate that identities like culture, religion, and belief are important aspects of one’s personality	3.47	.62
QG7	Gaining skills for sensitive practice within diversity requires a commitment to continuous professional development and lifelong learning	3.61	.62
QG10	Each person has more than one identity, which interlink and have a different meaning for each person	3.65	.49
QG11	I can identify intercultural and interreligious differences	3.23	.76
QG13	My culture and/or religion should not be informants of my perception of what is appropriate or inappropriate	3.58	.64
QD1	I interact with others with respect	3.54	.58
QD3	I can adapt my communication style and methods to better work with people from different cultures and/or religions, if need be	3.65	.49
QD6	I can practice in a respectful way toward others’ cultures and religions	3.69	.47
QD10	I have knowledge of a variety of communication methods and other skills that enable me to practice effectively with people from varied backgrounds	3.27	.78
QD11	I am fully able to identify biased attitudes or implicit assumptions of mine and will not act based on those	3.46	.81
QD12	I appreciate the differences within a single culture or religion, and I will avoid generalizing my knowledge but apply it separately for each person	3.35	.69

Following an interpretive analysis^6^ of the correlations, four distinctive areas are highlighted: decision-making, respect, nonjudgemental practice, and adaptability. To highlight these, a positive correlation was found between participants’ view that self-awareness is key in avoiding biased practices (*r* = .447; *P* < 0.05), as well as developing new skills to respectfully treat others in their practice (*r* = .375; *P* < 0.05). Further, a positive correlation at the 0.01 level (two-tailed) was found between self-awareness and recognition of own biases and appreciation of diversity (*r* = .452), as well as the likelihood of generalizations and exercise of microaggressions (*r* = .507). Equally important is the relationship found between the statements regarding high levels of self-understanding and respect (*r* = .648; *P* < 0.01), appreciation (*r* = .511; *P* < 0.01), and adjustable communication skills (*r* = .383; *P* < 0.01) toward diversity. Also, worth noting is the positive relationship between recognition of individual identities as unique and adaptability to ensure person-centered practice (*r* = .702; *P* < 0.00).

Further regression analyses highlight the main predictors for increased sensitive practice (Table 4). Specifically, the following are recognized: consciously avoiding generalizing across and within diverse cultural and religious groups, appreciation of diversity and the ability to identify intercultural and interreligious differences, as well as the conscious effort to avoid making decisions in practice based on own culture and/or religion.

**Table 4.** Regression Analysis.

Dependent variable	R	R square	Adjusted R square	Std. error of the estimate
Self-awareness of own biases (QE5)	.742^a^	.551	.514	.509
Own beliefs influencing (subconsciously) professional judgment (QE6)	.662^b^	.438	.398	.508
Sensitive practice skills require commitment to CPD and lifelong learning (QG7)	.655^c^	.429	.407	.422

^a^ Predictors: consciously avoiding generalizing across and within diverse cultural and religious groups (QG4 and QD12).

^b^ Predictors: QG4 and QG11.

^c^ Predictor: QG13.

## Discussion

**Figure 1.**
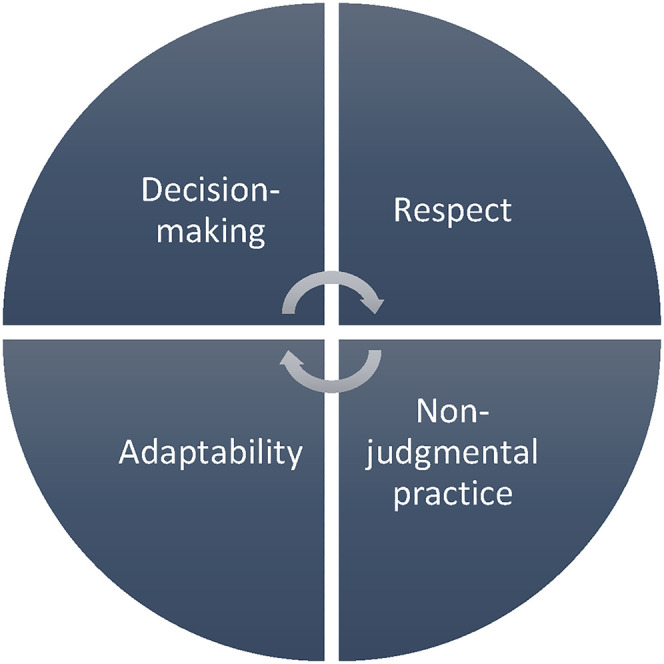
Qualities of culturally and religiously sensitive practice.

This study revealed the intersection of qualities that may enhance culturally and religiously sensitive end-of-life care practice. Figure 1 shows those qualities and their interconnections, while this section discusses those and the prerequisites for enhancing them and, therefore, ensuring sensitive practice.

Previous studies, as shown below, have identified ways in which cultural sensitivity in practice with specific populations can be improved. The findings from this study appear to corroborate with those from other studies, when considering other populations, yet this study’s original contribution is both culturally and professionally specific to Cyprus.

Respect is a key component when practicing with a culturally and religiously diverse population.^7,8^ Of course, this underpinning requirement to develop sensitivity toward diversity is neither new nor exhausted in practice and policy debates. An area of key concern in the UK, for example, has been the care of Muslim patients in a Christian environment.^9^ In their study, Lawrence and Rozmus^10^ opined that respecting Muslim patients’ practices and routine (eg prayer times) is of great importance to build stronger relationships and, consequently, practice with an element of cultural sensitivity. Similarly, Maguen et al.^11^ argued that working with cultural sensitivity with transgender people requires the development of accurate knowledge of terminology applicable to one’s identity, as well as an understanding of it. The principle of respect toward difference to improve culturally and religiously sensitive practice is found in the present study, too.

Further, the current study shows that decision-making processes and the ability to practice without biased judgment are both of significance when attempting to increase levels of sensitivity in practice. King et al.^12^ argued that effective culturally sensitive practice is that which promotes positive and strong relationships with patients, increased engagement of patients in decision-making and practice, as well as practice that is welcoming to family members and friends of the patient. As this study showed, acknowledgment of own biases and recognition of ways to manage practice without being influenced by personal values that may distort a patient’s experience, are all important to improve both culturally and religiously sensitive practice. This appears to be a transactional approach to becoming sensitive toward diversity, one that has been argued in learning previously. Scholes and Moore^13^ argued that transcultural knowledge exchange programs are an accurate and effective model for developing culturally sensitive carers. In their program, they engaged nursing students with exchange programs among three Institutions across England, the Netherlands, and Spain. Part of the program was the need for learning a new language and receiving the knowledge of others’ cultures. The outcomes of this program were telling about the changes in the levels of sensitivity measured among the students.

Further, the concept of adaptability is recognized as necessary when seeking improvements in culturally and religiously sensitive practice. Luper’s^14^ philosophical take on death and dying gives us a great context in which to understand adaptability in relation to diversity. In palliative and hospice care, it is important that one’s experience is perceived uniquely and not in relation or by comparison to others. Drawing on Luper’s work, the attempt to appreciate the unique nature of death and dying for those experiencing it, one has to be adaptable and adjust their sense of understanding to the epistemological concerns of the patient.

All four key components of what might promote culturally and religiously sensitive practice require development in three domains; avoidance of generalizations, appreciation of intercultural and interreligious difference, and nonbiased professional judgment. While the latter has been discussed above, it is worth noting the other two. First, this study shows that the less tendencies professionals have to generalize knowledge across a culture or religion, the more likely it is that their practice will be sensitive. Closely related to this is the second point made; to eradicate the tendencies for generalizations, the high risks of damage with labeling and stereotyping, appreciating further intercultural and interreligious diversity is important. In other words, the world is faced with cultural and religious diversity, but with cultural and religious plurality as well. There is diversity within diversity and the need for comprehending the subjective nature of how one acts out their culture and/or religion, which subsequently shapes up experiences pertinent to their palliative and/or hospice care, is necessary to develop sensitivity in practice.

## Limitations of the Study

This study is not without limitations. The underdeveloped areas of palliative and hospice care in Cyprus (Greek Cypriot controlled southern two-thirds of the island), as well as the lack of an established relationship between this area of practice and health care altogether, may be problematic when situating these findings. The researchers are mindful, as well, that most palliative and hospice professionals in Cyprus are providing cancer patient care predominantly. This limits the generalizability of the data. Further, the four qualities of sensitive practice identified in this study directly derive from the explorations about self-reflection, self-awareness, and self-critique among hospice and palliative professionals in Cyprus. That said, the findings are linked with the notion of working with people toward to end of their lives or while grieving. In other words, albeit the generalizability of the findings to medical and clinical practices altogether, this study can only claim the findings’ relevance to hospice and palliative care. Finally, the study reports on findings from professionals in the field. This, albeit important, reflects a level of self-perceptiveness. Future research will need to focus on collecting data from service users/clients/patients and family members/friends to develop a more rounded understanding in this area.

## Conclusions

Overall, this study verifies other studies’ findings and concomitantly is unique for it is culturally and professionally specific as it focuses on Cypriot professionals working mostly with cancer patients. This study reflects on the main factors effectively affecting and developing cultural and religious sensitivity in professionals’ practice in hospice and palliative care. It has identified that self-awareness, development of new skills to treat others, respectively, appreciation of diversity, recognition of individuals’ identities as unique, and adaptability to ensure person-centered practice is highly significant when applying sensitive practice with diverse patients. In conclusion, it is important to be noted that more training—deeper understanding of cultural and personal narratives and identities, including our own—and professional supervision were both heightened by the participants of this study as both needed to further develop their skills and self-awareness for the treatment of diverse patients. As Pentaris and Christodoulou^5^ stated, professional supervision enhances self-awareness and is a significant component to a more culturally and religiously sensitive practice in palliative care and hospice.
